# Supporting aboriginal knowledge and practice in health care: lessons from a qualitative evaluation of the strong women, strong babies, strong culture program

**DOI:** 10.1186/s12884-015-0433-3

**Published:** 2015-02-05

**Authors:** Anne Lowell, Sue Kildea, Marlene Liddle, Barbara Cox, Barbara Paterson

**Affiliations:** Research Centre for Health and Wellbeing, Charles Darwin University, Ellengowan Drive, Darwin, Northern Territory Australia; Midwifery Research Unit, Mater Research Institute, School of Nursing and Midwifery, University of Queensland, Brisbane, Queensland Australia; Strong Women, Strong Babies, Strong Culture Program Coordinator, Department of Health, Northern Territory Government, Darwin, NT Australia; Department of Health, Northern Territory Government, Darwin, NT Australia

**Keywords:** Maternal and child health, Indigenous health, Aboriginal culture, Intercultural health care

## Abstract

**Background:**

The Strong Women, Strong Babies, Strong Culture Program (the Program) evolved from a recognition of the value of Aboriginal knowledge and practice in promoting maternal and child health (MCH) in remote communities of the Northern Territory (NT) of Australia. Commencing in 1993 it continues to operate today. In 2008, the NT Department of Health commissioned an evaluation to identify enabling factors and barriers to successful implementation of the Program, and to identify potential pathways for future development. In this paper we focus on the evaluation findings related specifically to the role of Aborignal cultural knowledge and practice within the Program.

**Methods:**

A qualitative evaluation utilised purposive sampling to maximise diversity in program history and Aboriginal culture. Semi-structured, in-depth interviews with 76 participants were recorded in their preferred language with a registered Interpreter when required. Thematic analysis of data was verified or modified through further discussions with participants and members of the evaluation team.

**Results:**

Although the importance of Aboriginal knowledge and practice as a fundamental component of the Program is widely acknowledged, there has been considerable variation across time and location in the extent to which these cultural dimensions have been included in practice. Factors contributing to this variation are complex and relate to a number of broad themes including: location of control over Program activities; recognition and respect for Aboriginal knowledge and practice as a legitimate component of health care; working in partnership; communication within and beyond the Program; access to transport and working space; and governance and organisational support.

**Conclusions:**

We suggest that inclusion of Aboriginal knowledge and practice as a fundamental component of the Program is key to its survival over more than twenty years despite serious challenges. Respect for the legitimacy of Aboriginal knowledge and practice within health care, a high level of community participation and control supported through effective governance and sufficient organisational commitment as well as competence in intercultural collaborative practice of health staff are critical requirements for realising the potential for cultural knowledge and practice to improve Aboriginal health outcomes.

## Background

The gaps between Aboriginal and non-Aboriginal MCH outcomes in Australia are well known with a national *Close the Gap* campaign aiming to significantly reduce Aboriginal disadvantage [1,2]. Despite the increased focus on improving outcomes, the proportion of low birth weight babies born to Aboriginal mothers during the period 1991 to 2008 increased by 13% and is twice as high as non-Aboriginal mothers. There was also a significant increase in the rate difference of low birthweight babies born to Indigenous and non-Indigenous mothers (19%) over the same period [3].

The Northern Territory (NT), one of Australia’s eight jurisdictions, has some of the poorest maternal and perinatal morbidity and mortality rates in Australia with high rates of underlying diseases, reflecting poor living conditions and general poverty [4,5]. The NT is a vast geographic territory (1,353,201 square kilometres) with a small, highly dispersed population (n = 211,945), of which 26.8% are Aboriginal and Torres Strait Islanders [6]. The majority of Aboriginal people live in remote or very remote locations (79.0%) [7]. Aboriginal cultures and languages are strong and diverse across the NT where more than 100 languages are spoken and many people speak several of these languages [8]. The NT has two geographical regions – the northern region commonly referred to as the ‘Top End’ and the southern region known as ‘The Centre’ referring to Central Australia.

One approach to addressing some of the MCH challenges in the NT was the development of the Strong Women, Strong Babies, Strong Culture Program (the Program), which began as a pilot project in 1993 [9] in three Top End communities and later expanded to other communities in the Top End and the Centre. The Program evolved in recognition of the value of the cultural knowledge senior women can contribute through their participation in activities to improve MCH outcomes. The Program was developed in consultation with Aboriginal women and healthworkers and initially focused on poor nutrition and infections during pregnancy, with the aim of developing bicultural strategies to reduce the occurrence and effect of these conditions including lowbirth weight and preterm birth [10].

A quantitative evaluation of the Program in the three pilot communities compared birthweight, preterm delivery and trimester of presentation in pregnancy between the pre-Program phase (1990–1) and the post-introduction phase (1994–5) [9]. This study reported a statistically significant decline in low birthweight which was much larger (8.4%) than the decline in the rest of the NT (1.5%) [9]. A later study found that the improvement in birth weight in the pilot communities was maintained over time but no significant change was found in other communities which had subsequently commenced the Program [11]. The authors suggested the difference in results might have been due to differences in implementation of the Program and levels of support provided to the workers and identified a need for in-depth qualitative methods to identify the strengths and weaknesses of the Programs in different communities. Although the study had focused on the benefits of the Program to pregnant women they also noted wider social and economic benefits, including the opportunities for employment and the ‘*recognition of the skills of Aboriginal people in tackling their own issues*’ [11].

A qualitative evaluation of the pilot project was also conducted in 1994 [10] and identified the ‘most outstanding outcome’ as the development of a *partnership* in which Aboriginal women became teachers of traditional knowledge to their non-Aboriginal partners. They also suggested that the project’s success may be attributed in part to its drawing out of a generative theme which *gave primacy to culture and cultural renewal as the well-spring of health* but ultimately the success was due to Aboriginal women in remote communities being *committed to participation and taking control* of the process [10].

Following the pilot phase, the Program was funded by the NT Department of Health for a total of 12 communities and grants were paid to community organisations (such as Community Councils) to support the Program. Funding was used to provide ‘top-up’ payments for women (usually two in each participating community) employed by the Community Development and Employment Project (CDEP – a payment of a similar amount to unemployment benefits) or to ‘top-up’ Centrelink (e.g. aged pension or unemployment) payments. Coordinators (always Aboriginal women) were employed by the Department to support the community-based Strong Women Workers. Initially two Program Coordinators were employed (one in the Top End and one in Central Australia), which later increased to two positions in each region. The number of participating communities has varied considerably over the years. The largest number of communities funded to run the Program in any one year was 14. However, it has not run consistently, or sustainably, in all communities in which it has been started. Grant funding ceased on 1 July 2008 and Strong Women Workers were transferred to direct employment through the NT Department of Health either on a part-time or full-time basis depending on the size of the community and the preference of the individual staff. Strong Women Workers are selected through culturally appropriate community consultation and are employed at different levels reflecting their individual levels of cultural knowledge.

The Program is administered and governed separately to local health services. Program staff provide support as well as cultural and mainstream health education to young and pregnant women and women with children. In some communities the Strong Women Workers are located in community health centres and in other communities they operate from other locations. Program staff work collaboratively with local and visiting health staff, develop work plans every two weeks and provide fortnightly activity reports. Senior Strong Women Workers provide cultural education to younger staff and all Program staff participate in a range of health-related training activities. These include one week workshops twice each year for all Program staff, regional workshops on topics such as chronic disease, sexual health and nutrition, as well as ongoing reciprocal training between Strong Women Workers and health staff located in, or visiting, their communities.

From the earliest stages of the Program the overall aims and objectives have remained fundamentally consistent although a particular feature of the Program is the flexibility to adapt to different community needs and priorities rather than adhering to a strict protocol [11] (see Program Overview). Activities include working with midwives and other health staff in the clinic, conducting health-related education sessions at schools, playgroups and other early childhood services, development of educational resources, ‘culture camps’ for young girls and women, and other cultural activities with young girls and mothers with infants and young children.

### Program overview

The Strong women, strong babies, strong culture program promotes improvement in the health of Aboriginal women and their babies. The program aims to:

- improve the health and well being of all mothers and their newborn babies
- strengthen the family unit and help bring back cultural practices
- prevent and promote early intervention of lifestyle illness and disease before, during and following pregnancy
- provide a healthier community for future generations.

The program recognises the traditional cultural approaches to parenting and lifestyle, supporting pregnant Aboriginal women and their babies through better diet, education and antenatal care, with the aim of increasing the birth weight of babies and improving early childhood development. The program relies on and supports senior women in participating communities to provide direct support to pregnant women and their families. The senior women encourage attendance at antenatal care clinics and provide advice on nutrition. Connections and support for involvement in cultural events are an important part of the program. This particular program is one that has a strong community development focus and potentially major health benefits to Aboriginal people. This has a long term outlook with lasting benefits rather than only treating immediate health problems [12].

In 2008, the NT Department of Health commissioned an evaluation to identify enabling factors and barriers to the successful implementation of the Program, and to identify potential pathways/models for future development of the Program across the NT. In this paper, we focus on the inclusion of Aboriginal cultural knowledge and practice as a fundamental component of the Program drawing on findings from the interviews conducted during the evaluation.

## Methods

A collaborative approach was used in which key stakeholders (including Strong Women Workers, interested community members, Departmental and other organisational staff) were involved in framing the methodology, providing their perspectives on the Program and verifying the emerging findings to shape the framework for future development of the Program. The Evaluation Team included two researchers from Charles Darwin University (authors AL and SK) who have extensive experience in collaborative research with Aboriginal people in remote communities. As well, a senior Department of Health staff member and four Aboriginal Program Coordinators participated as advisors but were not directly involved in data collection or analysis.

The evaluation (conducted from May to November 2008) employed qualitative methods, utilising data from a range of sources including published and unpublished reports; interviews with past and present Program Coordinators (n = 6) and Department of Health staff (n = 15); and interviews in selected communities with past and present Strong Women Workers (n = 15), staff of local organisations and community members (n = 40) with interest in the Program. A total of 76 people were interviewed including Strong Women Workers from all but one currently participating community. Consent was obtained from all participants with the assistance of an interpreter when needed to ensure consent was genuinely informed. The option of recording oral consent on audio or videotape, when this was considered more appropriate by cultural advisors, was approved by the relevant Ethics Committees. Interviews were semi-structured, guided by discussion points relevant to each participant group relating to Program history, scope and activities, employment, resourcing and support issues, communication and ideas for future development of the Program. In-depth discussion of issues of particular relevance to individual participants was also enouraged and interviews were recorded on video or digital audio in the preferred language of participants, with a registered Interpreter when required. The opportunity for participants to use their own language in research is strongly advocated by Aboriginal researchers to ensure genuinely informed consent and enable in-depth sharing of their perspectives [13]. Interviews were conducted with individuals or with more than one participant, depending on their preference. Approximately 35 hours of interview data was recorded, translated and transcribed. Further informal discussions were also held with many of the participants to clarify or verify information at various times during the evaluation process.

Five remote communities were visited: four where the Program was currently operating and one where the Program had ceased to operate (three Top End communities and two in Central Australia). Communities were selected based on a purposive sampling strategy to maximise diversity in program history, Aboriginal culture and geographic dispersal; advice from Program Coordinators; and the communities’ ability to participate. The plan needed continual modification based on local context (availability of accommodation, illness of staff, cultural business and funerals) and not all of the planned community visits were possible. A qualitative data management program (ATLAS.ti) was used to assist with coding of data from all sources. An inductive approach was used in which categories of analysis were derived from the data and organised according to key themes. Emerging themes were further refined through an iterative process of discussion and verification with as many participants as possible, and modified as required. This process occurred informally throughout the period of data analysis and development of the evaluation report as well as at the annual Program workshops in both jurisdictions in which all the Strong Women Workers and Coordinators participated.

Ethics approval was received from the Human Research Ethics Committee (HREC) of the NT Department of Health and Menzies School of Health Research and the Central Australian HREC.

## Results and discussion

In the first section, we illustrate the role and importance of Aboriginal cultural knowledge and practice within the Program, particularly from the perspectives of Strong Women Workers. We then explore the complex and closely interrelated factors identified as sustaining and supporting the cultural dimensions of the Program as well as the associated challenges. Key themes to which these factors relate include: location of control over project activities, recognition and respect for Aboriginal knowledge and practice as a legitimate component of health care, working in partnership, communication within and beyond the Program, access to transport and working space, as well as governance and support.

### The role and importance of aboriginal cultural knowledge and practice

The Program was developed as a bicultural program intended to bring together Aboriginal and mainstream health knowledge and practice in improving MCH outcomes. In both the Top End and Central Australia Aboriginal cultural knowledge and practice have been considered fundamental components of the Program since its inception and this is consistently reflected in Program documents. Although mainstream health information is frequently integrated into Program activities, the cultural aspects of the Program are considered of central importance by Strong Women Workers and Coordinators from all regions, as well as many other participants. The role and value of Aboriginal cultural knowledge and practice within the Program and the critical influence of time and place are illustrated in the following sections.

#### Traditional health knowledge and practice

In all participating communities cultural education and traditional Aboriginal health practices continue to play an important role in the lives of Strong Women Workers, both as part of Program activities as well as beyond formal hours of work. In Northeast Arnhemland ‘*raypirri*’, part of the Yolŋu (local Aboriginal) system of cultural education provided by senior family members from birth through childhood and beyond, is central to the conceptualisation of the Program in this region. In Central Australia a similar concept is described as ‘Grandmother’s Law’ referring to cultural dimensions incorporated into the Program. The inherent flexibility of the Program to respond to local needs and priorities results in considerable variability in Program activities as illustrated in the following examples.

An important and widely recognised part of the Strong Women Workers’ role is to conduct traditional ceremonies with mothers and babies. Most health staff with any experience of the Program are aware of the ‘smoking ceremony’ that is conducted by Strong Women Workers with mothers and babies, preferably as soon as possible after birth. It is considered very beneficial for the health of both mother and baby:

*[we] take them out and smoke them and bush medicine .... helps to make the baby strong and the mother … it is very important - otherwise they’ll feel sick and feel sore when they get from hospital, and [it will] make the baby grow strong and healthy.* (Strong Women Worker)

However, awareness of the ‘smoking ceremony’ is often the extent of non-Aboriginal staff’s recognition of the cultural scope of the Program. Much of the work is ‘invisible’ to those outside the Program as it occurs away from the health centre, outside standard working hours and is conducted in local languages. For the Strong Women Workers the ‘smoking ceremony’ is just one very important part of a broad and continuous cultural process of promoting health and wellbeing that occurs throughout life. Education about, and provision of, traditional medicines is also important for scabies, toothache, infertility and various other uses. Some Strong Women Workers teach children in schools about traditional medicine and some also supply medicine to health centres, more commonly in Central Australia.

Although it is not documented as a formal part of their work, a number of Strong Women Workers also continue to assist women who give birth in their communities. Despite Departmental policy, which encourages women to travel to large centres for childbirth, babies are still occasionally born in communities. It is an area of their expertise that Strong Women Workers talk about with great enthusiasm and of which they are clearly proud. They also express frustration that this expertise is often not recognised, and opportunities to utilise their knowledge are limited.

A number of the Strong Women Workers practised as traditional midwives in the past when most women gave birth in their own communities and they recounted their experiences and shared their knowledge about birthing in great detail. The women shared knowledge relating to early identification of women who are pregnant. They described the traditional medicine and rubbing techniques used to relieve pain during labour, strategies for managing a breech birth and other difficult births, and appropriate ways of dealing with the placenta and umbilical cord. Some described sharing their knowledge with non-Aboriginal staff and some Strong Women Workers felt strongly that non-Aboriginal health staff should observe their skills to learn and recognise their knowledge. One of the non-Aboriginal health staff who has witnessed the Strong Women Worker assisting with childbirth gave her opinion:

*… you haven’t seen anything until you’ve seen [the Strong Women Worker] with someone in labour - she is stunning.* (Remote Health Centre Staff Member)

The same Strong Women Worker explained how she feels about assisting with birthing:

*…they should give that work to Yolŋu - we know how to take care of the women. Why are Balanda* (non-Aboriginal people) *frightened? Later on, when a woman gives birth outside we will be helping her. We are good at looking after the woman during birth. We know how to do that work. We can take the placenta out, we know how to cut the cord, we know how to wrap the baby and how to clean the mother properly. Wash the baby and wash the mother. Next morning we go for the smoking ceremony, take them for the smoking. It’s good if the Balanda can see what we are doing, observing our work….* (Strong Women Worker)

In both Central Australia and the Top End, women stress the importance of key female family members being involved with young mothers before, during and after the birth to educate and support them. The exclusion of these key senior women from the birthing process is considered to have serious consequences for the health and wellbeing of both mother and baby. Another consequence of birthing away from the community is the loss of the knowledge of traditional midwives. Most of the women with this expertise are now elderly and are concerned that they have little opportunity to pass on their knowledge to younger women.

#### ‘Going out’ – the importance of time and place

In all participating communities the importance of ‘going out’ was repeatedly emphasised and working away from the health centre was considered essential for the Program to be effective. For example:

*What help do we want? To go outside to work. Go out and work out there while other people watch how we work as a Strong Woman. Go and tell stories, go… My feeling and my thinking is why should I be there at the clinic all the time until my knowledge will fade away?* (Strong Women Worker)

‘Going out’ is considered important for a number of reasons. Not only is it necessary to exercise cultural knowledge and practice, it is also seen as an important opportunity for pregnant women to exercise themselves:

*…when they’re pregnant we take them out bush and help them out for bush tucker - and activities so they walk around - they have to walk around to find bush tucker, bush goanna and bush potato and porcupine…* (Strong Women Worker)

‘Going out’ is also important so education can be conducted in a culturally appropriate context, away from men. However, taking women and young girls out to an appropriate place often requires more time than the hours of formal employment allow and is sometimes not recognised as part of the formal paid work:

*In the afternoon when we have nothing to do we go out with those young girls, young mothers with the little ones. But we don’t get paid for that…* (Strong Women Worker)

One of the strategies for supporting intensive cultural education and practice – with sufficient time and in an appropriate environment is the implementation of ‘culture camps’. Despite considerable challenges in conducting camping trips over a number of days in a remote area - accessing transport and bad roads for example –– such camps are seen as an important and effective strategy for education and support for women and young girls.

The benefits for nurses working with community-based workers such as Strong Women Workers outside the clinic were also identified:

*I think the power shift that happens, naturally happens with minimal efforts, when nurses have to work outside the clinic, maximises the chances of engagement. By challenging that subtle power difference… if you work together within family groups, out of the clinic, you will be much more effective than just having a nurse and a health worker and baby room, nurse and health worker in a women’s room…* (Outreach Child Health Nurse).

Although the extent to which cultural knowledge and practice is included in Program activities varies, in all participating communities Strong Women Workers and others who participated in the evaluation strongly valued this aspect of the Program. However, this core feature of the Program appears to have diminished to varying degrees. In many communities there appears to have been a shift in emphasis from a focus on Aboriginal cultural knowledge and practice to a focus on mainstream health knowledge and practice as well as context of work, for example, working with the midwife in the clinic rather than with the women in the community or ‘out bush’.

### Sustaining and supporting aboriginal knowledge and practice within the Program

In the following sections we explore the factors that seemed to enable the inclusion of cultural knowledge and practice within the Program, as well as the challenges, related to a number of key themes.

#### Location of control over program activities

Participation in, and control over, Program activities by Aboriginal women in remote communities was a central principle of the Program from its inception [10], but has been limited by a number of factors throughout the life of the Program. A former Coordinator in Central Australia described the philosophy and practice of the Program during her involvement:

*…community based: senior women from community had control and ownership of the Program - that was the important thing, that’s why it worked but the downfall was that they needed that support… senior women didn’t have literacy…but they had the other speciality stuff - that community-based level and community participation and acknowledgement and acceptance of the Program and participation… I had no power when I was on the community - I just went with the flow… we worked as a team - showed them but they had to participate and do it themselves… the structure was good… it was a unique Program because [the Department] wanted to see if the concept of community control and ownership would actually work.* (Former Program Coordinator)

Location of control in the context of remote Aboriginal communities is of crucial importance to achieving and maintaining participation of community members:

*People have got to hold on to something that they own and that they control - it’s like hope, it’s like a lifeline thing. There’s chaos around people and they lose that little bit of independence, they’ll just give up.* (Previous Strong Women Worker)

Differences between the goals of the Program and the priorities of the Department, and of other organisations, emerged as one factor contributing to the variation in control over Program activities. For example:

*The … Program is supposed to be about resourcing Aboriginal women to do Aboriginal women’s business and look after these girls… There has been a creep from when it first started to make it meet Health Department agendas….* (Department of Health staff member)

Different expectations about the role of cultural expertise within health care influenced the location of control over Program activities. Even when the cultural knowledge of Strong Women Workers is recognised and valued by others, it is often viewed as a means to meet the priorities of the mainstream health system more effectively rather than as a valuable tool to improve health and wellbeing in its own right.

#### Recognition of and respect for aboriginal knowledge and practice

The centrality of culture in the health of Aboriginal and Torres Strait Islander people is emphasised in the National Aboriginal and Torres Strait Islander Health Plan 2013–23 [14]. This Program evolved through a recognition of the value of the cultural knowledge Aboriginal women can contribute through their participation in activities to improve MCH outcomes in their communities. However, misunderstanding and, in some cases, absence of recognition and/or respect for cultural knowledge and practice by some health staff were repeatedly identified as a barrier to successful implementation of the Program. A previous Strong Women Worker described the change in relationship between the Program workers and health staff since the early days of the Program in her community:

*Now it’s different… in the past we had freedom; now it’s a prison, owning Strong Women, hunger for power.*

In some communities the Strong Women Workers are frustrated that they rarely have the opportunity to ‘exercise’ their traditional knowledge and skills related to nutrition, medicine and other areas of cultural education that they consider vital for young women:

*…in the past the Strong Women didn’t learn about the clinical work… now you can see the difference - they’re doing different work, it’s not true Strong Women work - the Strong Women work is to go out into the community*. (Previous Strong Women Worker)

A senior woman also expressed her concern about the way the Program was currently being implemented in her community, and the increasing reliance on the mainstream system with the diminishing role of Aboriginal knowledge:

*…because Balanda are taking over more responsibility for Strong Women at the clinic they are not doing ‘exercise’ outside - going out and doing all these kind of things [that] they should be - exercising the Yolŋu knowledge … They should be at the camp or out there giving Yolŋu knowledge, educating Yolŋu people - and Yolŋu women and young people are not understanding [at] this time because they are more depending on Balanda medicine, Balanda stuff…* (Senior Community Member)

In this community, change has occurred due to problems with access to transport for cultural activities and the expectation by some health centre staff that the Strong Women Workers should work primarily in the health centre with pregnant women, assisting the midwife to achieve their goals.

The shift in control over Program activities, away from the Strong Women Workers themselves, sometimes resulted in ‘agenda creep’ where the Aboriginal cultural dimensions of the Program lose emphasis as the knowledge and practice of the biomedical domain take precedence. A shift has also been reported beyond the Program with a change in governmental attitudes away from incorporation of traditional knowledge within mainstream health care [15]. This may be due in part to the increasing emphasis on evidence-based health care that privileges biomedical services as research relating to Indigenous knowledge and practice in health care is sparse [15-17]. Understanding and respect for the legitimacy of Aboriginal knowledge and practice as an independent component of health care, not just as a means to meet mainstream service priorities, is essential to support the cultural dimensions of the Program.

#### Working in partnership

An evaluation of the pilot phase of the Program identified the ‘most outstanding outcome’ as the development of a partnership in which Aboriginal women became teachers of traditional knowledge to their non-Aboriginal partners [10]. A process of ‘two-way’ learning allowed sharing of knowledge while maintaining respect for, and integrity of, individual expertise from each cultural domain. Effective partnerships remain one of the critical factors contributing to success in those communities where such partnerships have evolved and been sustained.

The ‘two-way learning’ model, although highly valued by some, does not occur consistently across all locations. High turnover of remote health staff and inadequate understanding about the philosophy and scope of the Program, as well as a lack of cultural competence (particularly skills in intercultural communication, awareness of cultural specificity of mainstream health services and an understanding of power relationships) emerged as important barriers to effective relationships. These factors often result in excessive and/or inappropriate demands on Strong Women Workers time and a weakening of the partnership approach.

In some communities such collaborative relationships have been highly successful and a major factor in Program success. Although such intercultural collaboration has been a common and valued practice within sections of the Department in the past, there is no institutionalised policy, practice or training support to ensure a collaborative approach is sustained in a context of high staff turnover. Successful relationships therefore depend primarily on the motivation of individual staff to develop their knowledge and skills for working effectively in an intercultural context. As a result, the development of collaborative partnerships in which a balance is maintained between both domains of knowledge is not consistently achieved.

This is evident at a community level where Strong Women Workers are often expected to assist health staff to implement their agenda. Although partnerships may be perceived to exist, the level of Aboriginal control identified as crucial in the pilot phase is not always maintained. Even when there is an apparently successful partnership in which the cultural knowledge of Strong Women Workers is highly valued, the balance of power within the relationship can still be a problem, unrecognised by health staff. In one community, for example, the importance of collaboration for improving clinical outcomes was clearly recognised by health staff:

*I personally think that we need a partnership for things to happen, for things to be effective - the Yolŋu person has all the culture but they don’t always have all the clinical knowledge, don’t always understand it but if you’ve got the clinical and the cultural and work together then you share the workload, yes, it’s more expensive… but it works… and that’s why this Program works because we work alongside each other and it’s not ‘you do this and I’ll do that’ but ‘what do you girls want to do?’ ‘what should we do today?’ ….* (Remote Health Centre Staff Member)

In the same community the Strong Women Workers recognised their role in the health centre as effective in assisting the midwife to achieve her goals. However, they were frustrated that they couldn’t do the work that they considered important such as taking women and children out hunting and for cultural education in an appropriate environment. Although the Strong Women Workers had a positive personal relationship with the midwife they were unable to communicate their concerns to her and she was unaware of the consequences of the imbalance of power in the relationship. Despite the frustrations due to their lack of control over their activities, the Strong Women Workers there still valued their work with health staff.

Inadequate education of health staff about the Program, to ensure expectations and engagement were consistent with the philosophy and scope of the Program, was a common concern. For example:

*Because of the problem with many in the Department not understanding what the Program was about, the philosophical basis of it, that it was to resource senior Aboriginal women to do what they thought was necessary to get the girls to have decent sized babies…. as it [the Program] grew the problems started to arise. And no - there was never any proper orientation, it’s never been something that was explained to people, it was just this wonderful Program that was going to save the world.* (Department of Health staff member)

Lack of recognition of local cultural strengths by other health staff is another concern:

*… because the problem with nurses - they don’t know what they don’t know, so our challenge is to help them understand…the Aboriginal world view, this lovely connection to the family and who you are in the world and that it’s not just the absence or presence of disease, but it’s actually a much broader concept because maternal and child health is - should be -within a wellness model… so actually that is supported by the Aboriginal world view so much better.* (Previous Outreach Child Heath Nurse)

The need for health staff to develop specific skills in working collaboratively was also identified, for example:

*I also think the [health] team needs education on how to work with community-based workers because we are given information in our orientation [about the Program]… but that is not on the ground stuff…. We need to be encouraged as team members to work together more as a team and go out with people when we can and meet the community-based workers in community…* (Department of Health staff member)

Although improving the cultural knowledge of health staff is considered important this should not diminish the role of Strong Women Workers and other community-based workers:

*I think that was the biggest challenge is how to increase the expertise of medical staff without diminishing the expertise of Aboriginal everything, the way they think, the way they act, their right to be in the health centre, their right to be involved in the care of their own people, their right to be recognised as integral to that connection, because I think it’s so arrogant to think you can actually have any success in engaging Aboriginal people without that cultural broker.* (Former Outreach Child Health Nurse)

However, the perceived value of Strong Women Workers as ‘cultural brokers’ has been one of the challenges in implementing the Program. Excessive demands and expectations from various service areas for assistance from Strong Women Workers is a concern for Coordinators and Strong Women Workers in all regions. The motivation of Strong Women Workers to address the wide range of needs in their communities can sometimes result in ‘overload’ when staff from other programs utilise Strong Women Workers to assist them with their work:

*… people within the Department and probably at the clinic - need to be aware that you don’t overload those people because most of the Strong Women Workers or community-based workers are generally strong women within their community I’ve found, and they’re on this committee and they’re on that committee - everybody knows them, and people when they fly in, they want that person, they need that person to go to help them out with this and that. And they get so overloaded…* (Previous Program Coordinator).

The expectation that the role of Strong Women Workers is to help other staff to achieve their goals and the associated ‘overload’ diminishes the potential for Strong Women Workers to achieve their priorities in sharing their cultural knowledge and implementing cultural practices to promote maternal and child health. Intercultural partnerships are complex and diverse and an understanding of the factors that influence their effectiveness is critical to their success [18,19]. A collaborative approach in which the integrity of individual expertise from each cultural domain is maintained requires an adequate level of cultural competence including skills in intercultural communication and understanding of power relationships.

#### Communication – within and beyond the Program

Limitations in communication contributed to confusion about the roles and expectations of the Strong Women Workes and influenced the quality of collaboration as the previous sections illustrate. Achieving effective communication is a major challenge in any Program that involves diverse cultural and linguistic groups and high staff turnover. Such challenges were both reported and observed between participants at all levels of the Program, from community-based workers through to management. All of the Strong Women Workers Workers who participated in the evaluation are multilingual and are fluent in at least one, and often a number of Aboriginal languages. None speak English as a first language and their levels of English proficiency ranged from extremely limited to reasonably fluent in conversational English. All of these women have a high level of Aboriginal cultural knowledge, but again their level of mainstream cultural knowledge is variable. Any engagement with others who do not share the same cultural and linguistic background brings a high risk of miscommunication, although this was often not recognised, or was underestimated, by many participants in interactions observed during the evaluation. The educational potential for many of the Strong Women Workers and other health staff could not be realised when the Aboriginal women were unable to share the extent of their knowledge, or to fully or sometimes even partially, understand the information being shared with them.

The influence of different cultural protocols on communication was another source of difficulty. For example, during the interviews some Strong Women Workers expressed concerns about various issues that they had not felt able to communicate to the relevant staff due to cultural restrictions on direct confrontation, criticism or disagreement. Again, cultural competence training for health staff, particularly related to intercultural communication, is an essential strategy for improving engagement and effectiveness of services in Aboriginal communities. This is particularly challenging in a context such as the NT in which there is a high turnover of staff in health and other services engaging with the Program. High staff turnover is also a key influence on the effectiveness of intercultural engagement in other programs and clear and consistent communication about roles and expectations is particularly important in such contexts [20].

#### Practical barriers – access to transport and working space

Lack of transport is a serious barrier to effective implementation of the Program and a source of frustration for both Strong Women Workers and Program Coordinators. Lack of access to transport not only prevents Strong Women Workers from implementing their Program, but can also shift control over their activities to others. In some communities, the Strong Women Workers do have access to transport but only to meet the priorities of clinic staff, and their primary role is to pick up pregnant women for their antenatal check-ups and other ‘clinic business’.

The lack of working space for the Strong Women Workers, especially when their only option is to be located at the local health centre, also exacerbated their lack of control over their activities. In most locations the Strong Women Workers share a space with other programs or have no space at all in which to conduct education, store their resources, do their planning or write reports. The need for an appropriate working space to conduct education with young girls and pregnant women, safe from the intrusion of men, was repeatedly identified. Safe storage of resources, as well as a place to actually use them, a phone, fax machine and for some, a computer, were other needs not consistently met.

In one community the Strong Women Workers work in the midwife’s room when they are working with pregnant women but have no other area in which to conduct education or other program activities:

*…our office is at the laundry… We need our own office.* (Strong Women Worker)

In another community where the Program is based at the Health Centre, the Strong Women Workers are frustrated that the only place they have to do education with young girls and pregnant women is in the tea room.

Access to an independent working space was frequently identified as a critical factor to enable Strong Women Workers to maintain control and implement their Program successfully. Women in one Top End community described what they considered necessary for the Program to be effective:

*A place to work in, a shelter for women to tell women’s information in that men don’t enter. Somewhere far away, not close… A place to keep documents, a phone. A place where the older women can tell stories and the young women can record and write it down… a place run using Yolŋu methods.* (Strong Women Worker)

These key influences on implementation of the cultural dimensions of the Program – location of control over Program activities, access to transport, appropriate working space and expectations of health staff – were repeatedly identified by Strong Women Workers and Coordinators across all locations.

#### Governance and organisational support

Since the success of the initial project, the Department has presented the Program as an effective model that they strongly support. However, the inconsistency between this position and the inadequate funding provided for the Program to meet the perceived needs of Program workers was a source of concern both within and beyond the Department. For the first fifteen years of the Program small grants were provided to community organisations to administer the Program. Funding has substantially increased since the transfer of the Program to direct administration through the Department of Health, but recognition of the consequences of inadequate funding particularly in relation to the cultural dimensions of the Program, is important for informing future action.

An additional concern expressed by the Strong Women Workers in most communities related to the number of staff employed and level of pay they received. Consistently, Coordinators and Strong Women Workers argued that there is a need to have the option of more than two workers to respond to community needs and to ensure the full scope of the Program is implemented and sustainable. Employment of younger women, to learn from and assist the older women, was also considered important but often difficult to achieve. There was a strong consensus that a minimum of three to four workers is necessary to effectively implement and sustain the Program with the flexibility to offer a range of employment options (casual, part-time and full-time) to meet individual and community needs.

A crucial influence on the successful implementation of the cultural dimensions of the Program from its inception was the strength of both formal and informal Aboriginal governance with the employment (through the Department) of Aboriginal women as Program Coordinators to support the Strong Women Workers based in communities. The supportive and respectful relationships between the Strong Women Workers and Aboriginal Coordinators were identified as a key strength of the Program. A focus on empowerment of the Strong Women Workers is illustrated by one Coordinator in her description of what she considers to be important in her role:

*…giving women support out on the communities and helping them and working along with them to empower them… And they need to take control, that’s what needs to happen I believe.* (Program Coordinator)

The Coordinators are strong advocates for the Strong Women Workers and the Program with all stakeholders, have a deep understanding of the philosophy of the Program, and well-established connections with community members and Strong Women Workers. Many of the challenges described above impact not only on the Strong Women Workers but also on the Coordinators who have the additional demands of working at the interface between the communities and the Department. Sustaining an effective Program with sometimes very limited resources has been a continuing challenge for both Strong Women Workers and Coordinators. Their high level of commitment to the Program, despite these challenges, is evident in the number of staff who have continued to work in the Program for many years.

Under resourcing is a serious threat to sustainability and organisational commitment and provision of adequate resources is crucial to support the cultural dimensions of the Program. As well, the number of Strong Women Workers employed is often inadequate to meet the demands of an increasing workload due to, for example, increasing birth rates and the impact of high morbidity and mortality on individual workers and their families. Increasing Indigenous participation in the health workforce is considered imperative to improving health outcomes [21]. Increasing employment opportunities through the Strong Women Strong Babies Strong Culture Program is one strategy to achieve this.

In summary, the original philosophy and scope of the Program continues to receive strong support and there is an extremely high level of commitment from Strong Women Workers, Program Coordinators and many Department of Health staff, as well as extensive goodwill from other organisations and individuals. Two fundamental issues were identified through this evaluation: critical conditions for effective and sustainable implementation of the Program were often not met; and survival of the Program for more than twenty years, despite this, confirms the intrinsic value perceived by individuals, communities and the NT Department of Health. The findings presented in this paper also provide clear indications of the critical factors required to support sustained implementation of Aboriginal knowledge and practice within a mainstream health system (Summarised below).

### Enabling factors for supporting inclusion of Aboriginal knowledge and practice within a mainstream health care system

- Recognition of, respect for, and genuine support for cultural knowledge and practice reflected at organisation, system and staff levels in policy, funding, and staff development.
- Consensus and clear communication amongst all stakeholders regarding philosophy, scope of activities and roles of community-based workers and those that support them.
- A commitment to supporting a high level of Aboriginal participation and control at the community level through:
  - adequate funding for sufficient numbers of community-based staff, at a level that recognises their specific expertise;
  - provision of infrastructure required for effective implementation of Program activities; and
  - appropriate governance mechanisms that facilitate local control of Program activities, resources and funding.
- Effective collaborative engagement between community-based workers and other staff and organisations. This requires development of cultural competence of all staff engaging with community-based workers, including an understanding of power relationships, intercultural communication and collaborative practice.
- Flexibility to respond to different community needs and priorities over time and across diverse locations.

#### Limitations

The main limitation of the evaluation was the lack of time and funding to enable more extensive community feedback and verification of the findings. The evaluation coincided with the Australian Government Intervention and the disbanding of Community Councils which produced particular challenges influencing the timeline and approach. This also provided opportunities as the evaluation team held regular meetings to discuss emerging findings with Department of Health staff who were responsible for making recommendations on future community programs and employment options resulting from increased funding to remote communities.

## Conclusion

The Strong Women, Strong Babies, Strong Culture Program evolved through a recognition of the value of the cultural knowledge Aboriginal women can contribute through their participation in activities to improve MCH outcomes in their communities. Three crucial components of the Program were identified in a qualitative evaluation of the initial pilot project in 1994 [10]: the primacy of culture and cultural renewal as the’well-spring’ of health; community participation and control and the development of partnerships in which Indigenous women became educators of their non-Indigenous partner. Maintaining the strength of each of these components has presented substantial challenges in most communities since the inception of the Program.

The participation of community-based Aboriginal women in the Program and their control over Program activities is clearly crucial as is the support and advocacy role of Program Coordinators. Flexibility within the Program to allow for local adaption and development to meet specific and varying community needs is also important. The remarkable resilience of the Program in continuing to operate for more than twenty years despite serious challenges to implementation and sustainability and the high level of commitment of the Strong Women Workers and Coordinators suggests that the fundamental goals and structure of the Program continue to resonate with the aspirations and priorities of Program staff and communities. The recognition of, and respect for, Aboriginal knowledge and practice within health care, and recognition of their contribution to improving health outcomes, remain key features of the Program philosophy. However, the extent and ways in which these elements are realised in practice are influenced by a number of factors that need to be considered in any attempts to integrate cultural knowledge and practice with inmainstream health care system. These factors include recognition of, and respect for, Aboriginal knowledge and practice as a legitimate component of health care; a high level of community participation and control supported through effective governance and sufficient organisational commitment; and competence in intercultural collaborative practice of health and other staff to ensure effective engagement. All are critical requirements for realising the potential for cultural knowledge and practice to improve Aboriginal health outcomes.

## Abbreviations

*NT*: Northern Territory
*MCH*: Maternal and Child Health
*Yolŋu*: Aboriginal people of Northeast Arnhemland
*Balanda*: term used by Yolŋu to refer to non-Aboriginal people
